# Point-of-care testing for *Toxoplasma gondii* IgG/IgM using *Toxoplasma* ICT IgG-IgM test with sera from the United States and implications for developing countries

**DOI:** 10.1371/journal.pntd.0005670

**Published:** 2017-06-26

**Authors:** Ian J. Begeman, Joseph Lykins, Ying Zhou, Bo Shiun Lai, Pauline Levigne, Kamal El Bissati, Kenneth Boyer, Shawn Withers, Fatima Clouser, A. Gwendolyn Noble, Peter Rabiah, Charles N. Swisher, Peter T. Heydemann, Despina G. Contopoulos-Ioannidis, Jose G. Montoya, Yvonne Maldonado, Raymund Ramirez, Cindy Press, Eileen Stillwaggon, François Peyron, Rima McLeod

**Affiliations:** 1 Department of Ophthalmology and Visual Science, The University of Chicago, Chicago, Illinois, United States of America; 2 Pritzker School of Medicine, The University of Chicago, Chicago, Illinois, United States of America; 3 Institut de Parasitologie et de Mycologie Médicale Hôpital de la Croix Rousse, Lyon, France; 4 Rush University and Medical Center, Chicago, Illinois, United States of America; 5 Lurie Children’s Hospital and Northwestern University, Chicago, Illinois, United States of America; 6 Northshore Hospital, Evanston, Illinois, United States of America; 7 Department of Pediatrics, Division of Infectious Diseases, Stanford University School of Medicine, Stanford, California, United States of America; 8 Palo Alto Medical Foundation *Toxoplasma* Serology Laboratory, Palo Alto, California, United States of America; 9 Department of Medicine, Division of Infectious Diseases and Geographic Medicine, Stanford University School of Medicine, Stanford, California, United States of America; 10 Department of Health Research and Policy, Stanford University School of Medicine, Stanford, California, United States of America; 11 Gettysburg College, Gettysburg, Pennsylvania, United States of America; 12 Department of Pediatrics (Infectious Diseases), Institute of Genomics, Genetics, and Systems Biology, Global Health Center, Toxoplasmosis Center, the Center for Health and the Social Sciences, CHeSS, the College, The University of Chicago, Chicago Medicine, Chicago, Illinois, United States of America; Johns Hopkins Bloomberg School of Public Health, UNITED STATES

## Abstract

**Background:**

Congenital toxoplasmosis is a serious but preventable and treatable disease. Gestational screening facilitates early detection and treatment of primary acquisition. Thus, fetal infection can be promptly diagnosed and treated and outcomes can be improved.

**Methods:**

We tested 180 sera with the *Toxoplasma* ICT IgG-IgM point-of-care (POC) test. Sera were from 116 chronically infected persons (48 serotype II; 14 serotype I-III; 25 serotype I-IIIa; 28 serotype Atypical, haplogroup 12; 1 not typed). These represent strains of parasites infecting mothers of congenitally infected children in the U.S. 51 seronegative samples and 13 samples from recently infected persons known to be IgG/IgM positive within the prior 2.7 months also were tested. Interpretation was confirmed by two blinded observers. A comparison of costs for POC vs. commercial laboratory testing methods was performed.

**Results:**

We found that this new *Toxoplasma* ICT IgG-IgM POC test was highly sensitive (100%) and specific (100%) for distinguishing IgG/IgM-positive from negative sera. Use of such reliable POC tests can be cost-saving and benefit patients.

**Conclusions:**

Our work demonstrates that the *Toxoplasma* ICT IgG-IgM test can function reliably as a point-of-care test to diagnose *Toxoplasma gondii* infection in the U.S. This provides an opportunity to improve maternal-fetal care by using approaches, diagnostic tools, and medicines already available. This infection has serious, lifelong consequences for infected persons and their families. From the present study, it appears a simple, low-cost POC test is now available to help prevent morbidity/disability, decrease cost, and make gestational screening feasible. It also offers new options for improved prenatal care in low- and middle-income countries.

## Author summary

Toxoplasmosis, a disease caused by the parasite *Toxoplasma gondii*, presents a major health burden in both the developed and developing world. Untreated congenital toxoplasmosis causes damage to the eye and brain, but early detection and treatment reduce transmission and disease. Fetal infection can be promptly diagnosed and treated and outcomes can be improved. Gestational screening for toxoplasmosis has international precedent. In this paper, we demonstrated that the new *Toxoplasma* ICT IgG-IgM test had 100% sensitivity and specificity in detecting *Toxoplasma* infection (N = 180 U.S. sera from uninfected persons and those with varying parasite serotypes). The use of an inexpensive, easy-to-use point-of-care test facilitates screening of pregnant women for *T*. *gondii* infection. In turn, this facilitates prompt treatment for the infection and thereby reduces the health burden caused by this disease. This provides an opportunity to improve maternal-fetal care by using approaches, diagnostic tools, and medicines already available.

## Introduction

Toxoplasmosis, a disease caused by the Apicomplexan parasite *Toxoplasma gondii*, remains a major source of morbidity and mortality in the United States and globally. It causes a wide range of clinical manifestations, varying from a self-limited minor illness to devastating eye disease, congenital infection, and meningoencephalitis [1–4]. Treatment is currently imperfect, with available medicines effective against the active tachyzoite stage but not the slower-growing bradyzoite stage. In 2012, an estimate of annual morbidity from congenital toxoplasmosis was made [5]. If this estimate is applied to a 10-year time period, there would be 1.9 million new cases of congenital toxoplasmosis globally, causing 12 million disability-adjusted life years (DALYs) [5].

There is renewed interest in this organism in light of the more widespread recognition that 2–3 billion people are infected with this parasite with the potential to reactivate and cause life-threatening disease when significant immunosuppression occurs in these individuals. In addition, there is increasing evidence about its role in chronic inflammatory changes, epilepsy, and possibly neurodegenerative and neurobehavioral disease in some cases. Recent, new compounds with documented efficacy against the latent, encysted bradyzoite life-stage, as well as effective vaccines for prevention of disease in mice, have potential to reach human trials in the not-so-distant future [6–10]. However, current patients continue to suffer profound effects from this parasitic infection. It has been well-documented that early treatment of infected mothers decreases risk of transmission to the fetus and severity of clinical disease and therefore improves clinical outcomes [11]. In several countries, including France, Austria, and Uruguay, mandatory screening for this infection during gestation has saved lives and reduced morbidity and mortality [11–14]. However, while serologic screening has been demonstrated to be cost saving in countries that constrain costs of testing and medications, there is no screening program in the United States [15, 16]. Definition of better strategies to prevent this neglected, rarely diagnosed, and thus often untreated or mistreated disease is needed.

As a result, we reasoned that development and validation of accurate, easy-to-use, and inexpensive point-of-care (POC) testing could help solve this problem. A novel POC test, the *Toxoplasma* ICT IgG-IgM (LDBIO Diagnostic, Lyon, France; LDBIO) presents a unique opportunity, as it has been found to be accurate for detection of infection in France [17], and it is both economical, at ~US$4–8 per test, and rapid, with results available within 20 minutes, and requires no machinery. The cost we were charged was $4 and the list price was $8. $4 is used in our considerations from here on. It has been found to be reliable with low antibody titer sera and reliable when testing sera during seroconversion of pregnant women. For example, it was sensitive and specific for sera from a pregnant woman who initially was seronegative, then had only IgM, and lastly had IgG and IgM specific for *T*. *gondii*. The test is being used in France and has been studied with a French cohort in Lyon, where it was found to be both sensitive and specific. However, as has been documented previously, the population of European *T*. *gondii* is more genetically uniform, with predominance of Type II strains, which is distinct from the parasites found in the Western Hemisphere. Congenital *Toxoplasma* infection in the United States is caused by a more eclectic group of parasites, with Type II, Type I-III, and Atypical parasites (non-II) causing disease. Given the genetic diversity that characterizes the U.S. population of *T*. *gondii*, we felt it was important to assess ease of performance, sensitivity, specificity, and positive and negative predictive value of the test for U.S. patients.

The National Collaborative Chicago-Based Congenital Toxoplasmosis Study (NCCCTS) cohort is well characterized, with sera stored from these patients evaluated across many decades. We tested these samples for parasite serotype [18]. Herein, we present the application of this novel POC testing to distinctly American sera with the goal of preliminary validation of its use for patients in the United States.

## Methods

### Obtaining serologic samples

Serologic samples were obtained from those in the NCCCTS cohort, as well as from volunteers. All seropositive persons had serum tested using gold-standard serologic testing in the Palo Alto Reference Laboratory previously with Sabin-Feldman Dye Test and IgM ELISA, with the exception of some seronegative volunteers, who were tested in the University of Chicago Hospitals CLIA-approved Clinical Laboratory, which currently uses a Bio-Rad assay [18, 19]. Mothers of congenitally infected children in the NCCCTS also had their sera tested with a peptide ELISA to determine II, NE-II (I/III) or I-IIIa, or Atypical (response to II = NE/II) serotype, as described earlier [18]. Among our samples, there were 13 sera obtained from mothers within 2.7 months of birth of their congenitally infected child. Seronegative persons were laboratory personnel and fathers or adoptive mothers of congenitally infected children (18 women, 33 men).

### POC testing specifics

The POC testing materials were purchased from LDBIO in Lyon, France (http://www.ldbiodiagnostics.com). Additional details concerning this test are described in Chapey, Wallon, and Peyron [17].

### Serologic sample testing protocol

Sera had been stored at −20 or −80°C prior to testing and were thawed completely. Using a micropipette, 30 μL of serum was removed and placed into the well of the POC test. Three drops of eluent (provided in a dispensing bottle for drops in the kit) were then placed in the well. All POC tests were interpreted after 20 minutes. Results were interpreted by two individuals blinded to whether the serum was known to be positive or negative or of a certain parasite serotype. They determined whether the test was positive (as indicated by a pink, positive line and a blue, positive control line on the test) or negative (as indicated by the absence of the aforementioned pink, positive line and the presence of the blue, positive control line). This confirmed interpretation.

### Comparison of POC results with conventional serologic testing

Once the samples had been interpreted to be positive or negative, the data were compared to records of serologic testing maintained for the NCCCTS cohort for the purposes of statistical analysis. Earlier serological testing had been performed and interpreted by the Palo Alto Medical Foundation *Toxoplasma* Serology Laboratory (PAMF-TSL) [20]. A standard formula for sensitivity, specificity, positive predictive value, and negative predictive value was used to determine test characteristics for these samples [21, 22].

### Cost comparisons

We calculated the cost of monthly prenatal screening throughout gestation for toxoplasmosis in the U.S. using the *Toxoplasma* ICT IgG-IgM test as a POC test in the OB/GYN healthcare provider office (that costs $4-8/test) versus a commercially available *Toxoplasma* IgG and IgM test (that costs $650 for the *Toxoplasma* IgG and IgM).

### Ethics

The NCCCTS was/is conducted with ethical standards for human experimentation established in the Declaration of Helsinki, with prior University of Chicago Institutional Review Board approval (University of Chicago IRB Protocols 8793, 8796, 8797, 15408A, and 16708A) and in accordance with Health Insurance Portability and Accountability Act regulations. It was reviewed regularly by a Data Safety Monitoring Board. Informed consents were obtained from subjects for all aspects of the NCCCTS, including collection of serum samples, in accordance with University of Chicago Institutional Review Board and National Institutes of Health guidelines. All adult subjects provided informed consent. For any participant under the age of 18 years old, a parent or guardian provided informed consent on behalf of the subject. All consent was written, except for some volunteers who were not part of the NCCCTS, who provided oral consent. These volunteers who provided oral consent did so decades ago, at a time when obtaining written consent was not a usual part of obtaining a serum sample. Oral consent was witnessed. All serum samples were obtained with IRB approval.

### Statistics

ANOVA was used to assess differences within groups with respect to time from the birth of an infected baby to the collection of serum samples. Given the tendency for duration data to be skewed, the results of ANOVA were confirmed with Kruskal-Wallis. P < 0.05 was considered to be significant for time from birth to sample obtained.

## Results

### Samples tested

Results are from the following 116 serum samples from chronically infected persons tested: 48 samples from patients infected with Type II parasites, 14 samples from patients infected with Type I-III parasites, 25 samples from patients infected with Type I-IIIa parasites, 28 samples from patients infected with Atypical-type parasites, and 1 sample from a patient who was not typed. Thirteen additional samples were from acutely infected persons (less than 2.7 months since birth of their congenitally infected child). These 13 acutely infected persons with serum tested at the time of their primary infection included: 5 samples from patients infected with Type II parasites, 1 sample from a patient infected with Type I-III parasites, 4 samples from patients infected with Atypical-type parasites, and 3 samples from patients infected with parasites of unknown serotype. 51 samples from seronegative persons also were tested.

### Comparison of *Toxoplasma* ICT IgG-IgM POC test versus reference *Toxoplasma* serologic testing

**Fig 1:**
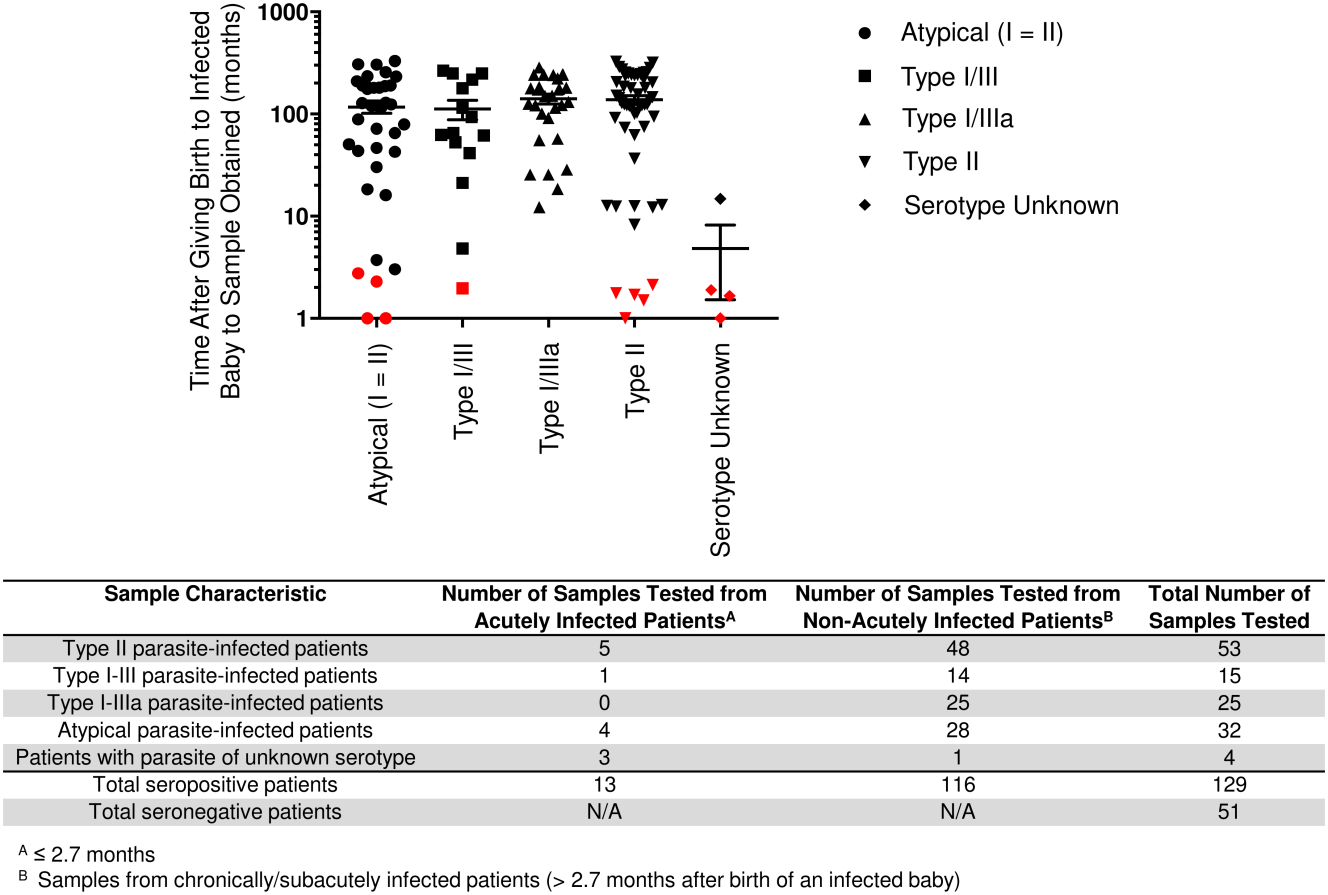
Chronically/Subacutely infected patients by parasite serotype as a function of time from birth of congenitally infected baby to sample obtained and seronegative patients. *Toxoplasma* ICT IgG-IgM test results and parasite serotype. Results were obtained using sera from chronically, subacutely, and acutely infected persons with differing parasite serotype as a function of time from birth of this congenitally infected infant to when the sample was obtained. These are results from sera that have been stored at varying times after visits of families to the NCCCTS. Acute sera were collected ≤2.7 months after the birth of the congenitally infected person and are shown with red symbols. Chronic subacute sera, shown with black symbols, were from 145 to 9500 days after the birth of the congenitally infected person. Almost all persons had been serotyped earlier in the study. There was one father tested and one congenitally infected adult. The serum samples, otherwise, were from mothers at the times from birth of the infected person. S1 Table presents these detailed data. This S1 Table also presents the mother’s serologic test results at the time of the congenitally infected person’s birth or in the case of congenitally infected persons missed at birth and presenting later in life (historical cohort) at the time of the first visit to Chicago. These data demonstrate functioning of the *Toxoplasma* ICT IgG-IgM with sera from parasites that have caused congenital toxoplasmosis in the U.S. They are not from pregnant women. This was already tested in France where this test has performed well. Duration from birth provides relatively precise time for the chronic infection in persons who have been carefully followed longitudinally, prospectively. The data presented had no statistically significant difference (P > 0.05) between time from the birth of a congenitally infected baby and obtaining the serum sample related to serotype, as determined by ANOVA (P = 0.59) and secondarily confirmed by Kruskal-Wallis (P = 0.52). This timing indicates that the *Toxoplasma* ICT IgG-IgM POC test is reliable in identifying acutely infected U.S. persons and subacutely and chronically infected U.S. persons even many years after infection. The 13 acute sera, ≤2.7 months from time of birth, were also positive. Mean and standard deviation are indicated. ^a^ In S1 Table, samples from chronically/subacutely infected persons (>2.7 months after birth of an infected baby); ^b^ Total includes samples from persons who are either acutely (≤2.7 months after birth of an infected infant) and chronically/subacutely infected.

Fig 1 and S1 Table show the time from the birth of a *T*. *gondii*-congenitally infected child to when the sample was obtained, organized by parasite serotype. S1 Table has the serologic test results for the mother at the time of diagnosis of her child that documents her seropositivity. This includes children diagnosed at birth. There were no false positives or false negatives. Test characteristics, including sensitivity, specificity, positive likelihood ratio, and negative likelihood ratio, are in Tables 1–3. The *Toxoplasma* ICT IgG-IgM test proved highly sensitive (100%) and specific (100%) in testing human sera from patients with infections with *T*. *gondii* strains circulating in the United States. There was no significant difference between samples based on serotype with respect to the time from the birth of an infected baby to sample collection by ANOVA (p = 0.59) and Kruskal-Wallis (p = 0.52).

**Table 1:** Summary of results with *Toxoplasma* ICT IgG-IgM test and reference tests: Summary of data used to calculate sensitivity and specificity.

		Reference Testing		
		Reference Positive^a^	Reference Negative^b^	Total
***Toxoplasma* ICT IgG-IgM Result**	**Positive**	**129**	**0**	**129**
	**Negative**	**0**	**51**	**51**
	**Total**	**129**	**51**	**180**

^a^ Based on gold-standard serologic testing at PAMF-TSL at earlier time when child diagnosed

^b^ Based on testing at PAMF-TSL or the University of Chicago Laboratory (Methods used included Vidas IgG, Bio-Rad IgG, bioMerieux Direct Agglutination, at various times)

**Table 2:** Summary of results with *Toxoplasma* ICT IgG-IgM test and reference tests: Subgroup breakdown.

Subgroups	Chronic/Subacute ^a^	Acute ^b^	Negative ^c^	Total
***Toxoplasma* ICT IgG-IgM Positive**	116	13	0	**129**
***Toxoplasma* ICT IgG-IgM Negative**	0	0	51	**51**
**Total**	116	13	51	**180**

^a^ > ≈ 2.7 months after the birth of an infected baby

^b^ ≤ ≈ 2.7 months after the birth of an infected baby

^c^ Based on testing at PAMF-TSL or the University of Chicago Laboratory (Methods used included Vidas IgG, Bio-Rad IgG, bioMerieux Direct Agglutination, at various times)

**Table 3:** Summary of results with *Toxoplasma* ICT IgG-IgM test and reference tests: Test parameters of *Toxoplasma* ICT IgG-IgM POC test.

Test Parameter	Result	95% CI
Sensitivity^a^	100%	97.18–100%
Specificity^b^	100%	93.02–100.00%
Positive likelihood ratio^c^	N/A (specificity 100%)	N/A (specificity 100%)
Negative likelihood ratio^d^	0.00	0.00

^a^ Sensitivity = TP/(TP + FN), where TP = number of true positives, FN = number of false negatives

^b^ Specificity = TN/(TN + FP), where TN = number of true negatives, FP = number of false positives

^c^ Positive likelihood ratio = (sensitivity)/(1 − specificity)

^d^ Negative likelihood ratio = (1 − sensitivity)/(specificity)

### Cost

Table 4 summarizes cost calculations for prenatal screening in the U.S. using the *Toxoplasma* ICT IgG-IgM POC test. In this table, we also contrast cost for standard anti-*T*. *gondii* IgM and IgG testing in a commercial laboratory at a university hospital. In Table 4, we considered what a person with indemnity insurance that paid in full in one U.S. hospital is charged. This information was provided by the hospital laboratory (Table 4). This is contrasted with reagent costs and the time for a medical assistant or technician to perform the *Toxoplasma* ICT IgG-IgM test. This analysis was with serum, or cost for whole blood collected from finger prick. This includes costs for materials, reagents, time for performing and interpreting the test and entering the result into the patient’s medical record (Table 4). We have previously shown that for the United States, at a cost of $12 per test and prevalence as low as 1/10,000 live births, monthly prenatal screening with treatment was found to be cost-saving [15]. This was without addressing costs of quality of life or suffering for families and patients, the prevention of which confers separate and profound benefit for a program with testing, accurate diagnosis, and treatment. A $4 point-of-care test ($4 was the charge we paid; $8 is listed on the website) increases the cost-savings over what was demonstrated in detailed cost-benefit studies for the United States and Austria [12]. In terms of charges actually billed, if the cost were $650 for testing once, as it is at present in a university hospital, then ten tests (that would be needed for monthly testing during pregnancy) would cost $6,500 for one pregnancy. This far exceeds the capacity of an obstetrician to function within his or her $1,000 capitation for providing care per pregnancy. However, at $40 per pregnancy for ten tests (that would be needed for monthly testing during pregnancy), this becomes a feasible opportunity to prevent, diagnose, and treat accordingly this infection and its consequences.

**Table 4:** Economic considerations for point-of-care test compared to hospital-administered test.

Test Type	Cost per Test (USD)	Cost per Pregnancy (10 Tests) (USD)	Cost for 100 Pregnancies (Estimate of an Obstetrical Practice) (USD)	Cost Savings on Testing Alone (USD)
Standard testing for *T*. *gondii* (at the University of Chicago Hospitals)	$650	$6,500^a^	$650,000	$0
Standard testing for *T*. *gondii* (estimate per Stillwaggon et al. [15])	$12	$120	$12,000	$638,000
*Toxoplasma* ICT IgG-IgM POC test with “bookend” multiplexed testing	$4–8	$40–80	$4,000–8,000^b^	$638,000–646,000^c^
*Toxoplasma* ICT IgG-IgM POC test with fingerprick on whole blood	$4.95^d^	$49.50	$4950	$645,050
*Toxoplasma* ICT IgG-IgM POC test with saliva	$5.55^e^	$55.50	$5550	$644,450

^a^ Well above standard capitation for pregnancy.

^b^ Initial investment for POC testing will also include BD Microtainer Tubes: US$38.25 for 50 tubes, Sprout Centrifuge: US$228, Class I biosafety cabinet: US$6,546; this cost is still lower than conventional testing and substantial cost savings remains.

^c^ Cost-savings could be many fold greater by multiplexing testing for HIV, syphilis, CMV, hepatitis B, herpes simplex, and potentially Zika virus or *Trypanosoma cruzi*, along with *T*. *gondii* IgG and IgM. This could reduce costs associated with individual testing. Additionally, monthly testing could enhance maternal-child healthcare by increasing interactions with physicians, promoting screening for pre-eclampsia and gestational diabetes.

^d^ This cost includes the cost of the *Toxoplasma* ICT IgG-IgM test as well as the cost of an individual lancet (estimated at US$0.33) and a Sarstedt Minivette POCT collection pipette (estimated at US$0.62); collection of whole blood obviates need for initial investment for centrifuge, electricity.

An extra 1 ml tube of serum obtained at the time of other tests and brought by the patient to a nurse’s aide or technician, in an outpatient setting who centrifuges the sample to collect serum, or collection of finger prick blood, takes 4–5 minutes of working time. Time as cost to place serum or obtain whole blood and buffer onto the *Toxoplasma* ICT IgG-IgM test strip and interpret results after 20 minutes (~5 seconds for each task after obtaining the blood) is included in those 4–5 minutes. Another 2 minutes is needed to enter the result in an electronic database like EPIC or less time if it is a hand written chart. This is the same procedure that is used in the obstetrics practice in the same hospital where the charge for the standard list costs for a patient with indemnity insurance was obtained. Total medical assistant or technician time per patient is ~6 minutes. Hourly wage (including benefits) for the technician in the same hospital is ~$20. Thus, ~6 minutes of his/her time is ~$2. Costs for tube, devices used for finger stick, alcohol wipe, gauze, Band-Aid would add another ~$0.25. Costs for time discussing results with the patient are the same in all scenarios as are costs for confirmatory testing when initial testing shows infection was acquired recently during gestation. Multiplexed nano testing reduces costs further, with first test at 11 weeks gestation and then at term. This reduces ambiguity about IgG and IgM seropositivity, facilitating avidity and other reference laboratory testing when needed.

^e^ This cost includes the *Toxoplasma* ICT IgG-IgM test and the cost of a saliva swab (Beaver-Visitec- #58109, US$0.79 each) and a swab storage tube (Salimetrics- US$0.76 each)

## Discussion

Herein, this novel *Toxoplasma* ICT IgG-IgM POC test for *Toxoplasma* IgG and IgM has proven very effective at identifying that sera of U.S. patients with known *T*. *gondii* infection are seropositive, and distinguishes them from those without serologic evidence of infection. It is also capable of identifying sera of acutely infected patients with high accuracy [17]. This new test has proven to be an effective screening method; it is accurate, rapid, and inexpensive. We were charged US$4 per test kit.

The novel POC test, the *Toxoplasma* ICT IgG-IgM test (LDBIO Diagnostic, Lyon, France; LDBIO), is already commercially available in France and represents a unique opportunity, as it has already been found to have a very good diagnostic performance when tested for detection of *Toxoplasma* infections from *T*. *gondii* strains circulating in France [17]. Moreover, it is both economical, at US$4–8 per test, and rapid, with results available within 20 minutes, and requires no large equipment. In the prior validation study in France, the *Toxoplasma* ICT IgG-IgM test was tested with 400 samples (99 positive for IgG and/or IgM and 301 negative for IgG/IgM when tested with the reference, widely commercially used, Architect automated chemiluminescence test). There were zero false negative *Toxoplasma* ICT IgG-IgM test results, while 13 false-positive *Toxoplasma* ICT IgG-IgM test results were identified among 301 seronegative samples. The *Toxoplasma* ICT IgG-IgM test correctly identified 21 positive sample that had only low IgG titers and also was reliable when testing sera during seroconversion of 5 pregnant women. Specifically, the *Toxoplasma* ICT IgG-IgM correctly characterized a pregnant woman who initially was seronegative by the Architect test (Architect negative; *Toxoplasma* ICT IgG-IgM negative), then had only IgM (Architect IgM positive, IgG negative; *Toxoplasma* ICT IgG-IgM positive), and lastly had IgG and IgM specific for *T*. *gondii* (Architect IgG and IgM positive; *Toxoplasma* ICT IgG-IgM positive) [13]. We did not encounter in testing the U.S. sera the few false-positive results found in France in the prior validation of the *Toxoplasma* ICT IgG-IgM test in France. A total of 580 sera were tested using the *Toxoplasma* ICT IgG-IgM POC test, i.e., in the present *Toxoplasma* ICT IgG-IgM validation study in the U.S. (180 sera), and in the prior *Toxoplasma* ICT IgG-IgM validation study in Lyon, France, by Chapey et al. (400 sera) [17]. This included in total 228 positive samples and 353 negative samples by reference *T*. *gondii* serologic testing.

In the U.S., there was 100% sensitivity and specificity, with perfect correlation with serologic status. In France, performance was also excellent, with 97% sensitivity and 96% specificity and documentation that seroconversion was detected in pregnant women in this commercially available test, already approved for clinical use in France. In France, there were 13 false positive sera among 109 true negative sera. There were 3 false positive nonspecific IgMs in true negative sera in France for these three reference Architect test results. In subsequent testing, these 3 women did not develop *T*. *gondii*-specific IgG. Thus, this test appears to be comparable to other standard, commercially available, conventional serologic tests, and it appears to detect infection with parasites found in France and in the U.S. Practically, for this combined IgG/IgM testing, a positive result would precipitate confirmatory testing [23]. Therefore, if any false-positive results did occur, they would be detected with the follow-up testing and not result in harm to the patient. As this test is used, it is a screening test, and patients should be informed that confirmatory testing will be necessary for a positive result.

**Fig 2:**
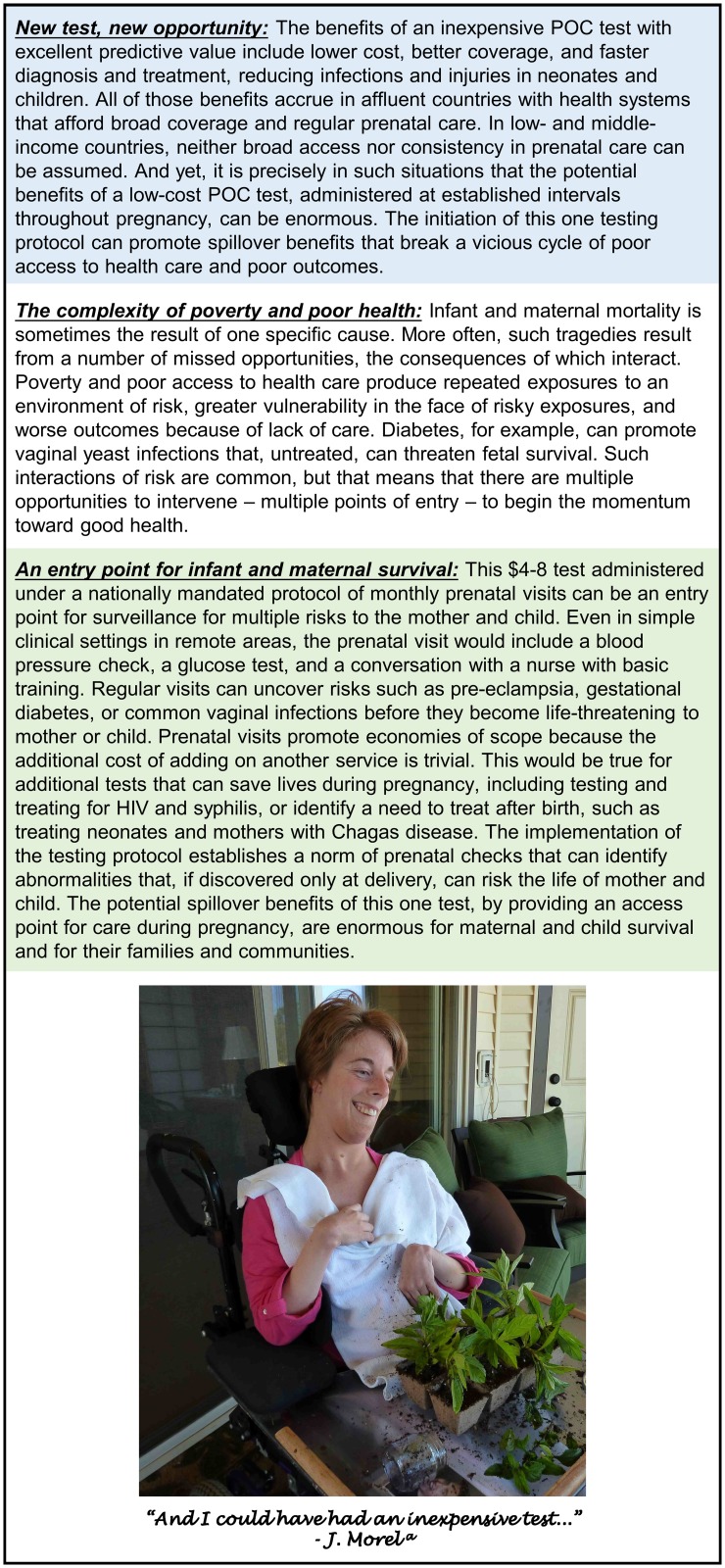
Gestational screening to save mothers’ and children’s lives and health care costs. Screening pregnant women for acquisition of *T*. *gondii* infection during gestation using inexpensive point-of-care tests will help in countries with limited resources as well as in countries that have abundant resources but do not have gestational screening programs, such as the U.S. ^a^ Photograph reproduced with permission.

Testing with such a POC test like *Toxoplasma* ICT IgG-IgM test could bring with it, especially in low- and middle-income settings, the incentive for ideal, more frequent monthly obstetrical care for pregnant women, improving maternal and child health. Additional cost savings and maternal-fetal health benefit could occur with multiplexed testing at the beginning and end of gestation. This could screen the patient not only for *Toxoplasma* infection, as with the *Toxoplasma* ICT IgG-IgM POC testing, but also for HIV, syphilis, hepatitis B, CMV IgG and IgM, herpes zoster, and immunity to rubella [24, 25]. Inclusion of other pathogens, such as Zika virus or *Trypanosoma cruzi*, the causative agent of Chagas disease, could also be beneficial in areas where those infections are prevalent. This multiplexed testing at the beginning and end of gestation, in combination with *Toxoplasma* ICT IgG-IgM POC testing on a monthly basis between the two multiplexed tests, could be remarkably cost-saving for patients and healthcare systems more broadly. It could facilitate profound improvement in quality of life and care for whole families. The considerations outlined in Fig 2 demonstrate many potential benefits from an inexpensive POC-based prenatal screening program and the “spillover” improvements in health care and outcomes that can result.

Of note, the test was not effective in the analysis of saliva and we were advised by the manufacturer that, in its present iteration, it was not reliable for testing whole blood when patients had low levels of antibodies, as the pink indicator was obscured by a yellow color from the whole blood (D. Limone, personal communication to R. McLeod, 2016). Preliminary tests showed it could detect antibodies in a few persons. However, further work to create a test with a dark-colored indicator is underway with future studies planned similar to those described initially by Chapey et al. for the *Toxoplasma* ICT IgG-IgM test when first tested in France (FP, PL) [17]. POC tests function well for other infections such as HIV. POC testing for *T*. *gondii* based on testing saliva would reduce the need for venipuncture or fingerstick.

The data herein with sera from U.S. patients infected with Type II, I/III, I/IIIa, and Atypical parasites demonstrate that the pink line indicator works well. The black indicator kit utilizes the same antigen and test strip so detection of infections with U.S. parasites would be comparable. We also wanted to develop a method whereby this test, proven herein to be very high-functioning, could be made a true point-of-care test with serum in a setting without use of a central laboratory. Thus, we developed a simple, practical method whereby this could be safely utilized in an obstetrical outpatient setting. Although more complicated than testing whole blood from a fingerprick, this approach makes the test feasible at very low cost following a small initial investment. This approach of obtaining serum in the clinic and testing is likely to be replaced in some settings with a test for whole blood currently in development, should it prove to be as sensitive and specific. Whole blood obtained by fingerstick, or saliva, is useful when processing of sera in a central laboratory is costly or inconvenient. Further, more extensive testing is needed for confirmation that this test with the dark colored indicator can be used with whole blood. This would not require electricity, centrifuges, or anything beyond a capillary tube, lancet, and the test kits at point of care.

**Fig 3:**
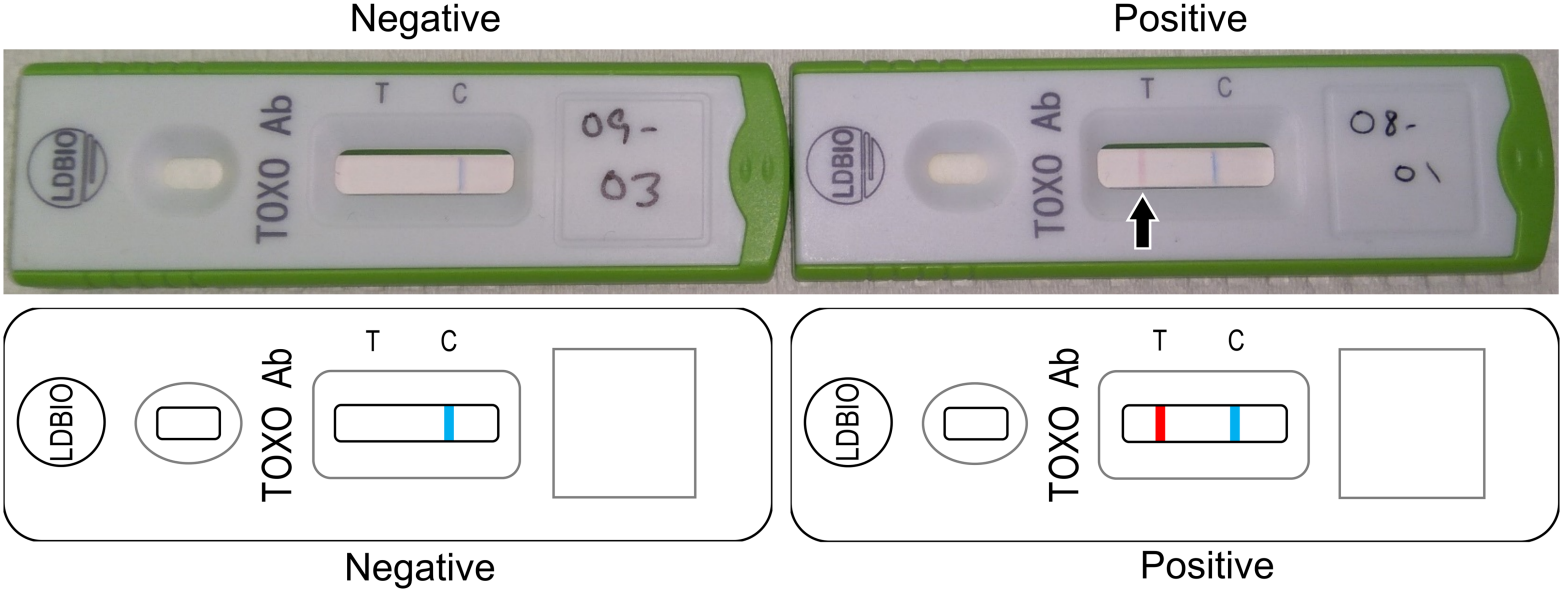
Implementation of *Toxoplasma* ICT IgG-IgM POC testing with separation of serum at point of care and representative *Toxoplasma* ICT IgG-IgM test negative and positive test results for sera. This involves a lancet to obtain the sample with fingerprick ($0.13 for the lancet and a very low cost for alcohol wipes), a small centrifuge tube to separate the serum, a small Class II biosafety cabinet ($6546) for safe handling of samples, and a small centrifuge for separating serum ($228), from which serum can be removed easily and be tested. The following methodology is described in Chapey [17]: Briefly: the *Toxoplasma* ICT IgG-IgM assay is based on a lateral flow chromatographic immunoassay (LFCI) technology that allows the simultaneous detection of *T*. *gondii* IgG or IgM antibodies in human serum/plasma [17]. A minimum sample volume of 30–50 μL of serum/plasma is required [17]. Each cassette contains: a) a nitrocellulose strip on which there are two reactive bands, one with the *Toxoplasma gondii* antigen (from whole cell lysate) called the “test” band (T band) and one with the rabbit gamma globulins called the “control” band (C band); b) a fiberglass support (conjugate pad) which is impregnated with red latex particles coupled with *Toxoplasma* antigens (“test” latex = T latex) and blue latex particles coupled with goat anti-rabbit IgG (“control” latex = C latex) [17]. The test is run by dispensing the serum/plasma and an eluting solution (eluent) in the “sample well” of the cassette [17]. With the addition of the eluent, starts the concomitant migration (chromatography) of the serum/plasma and the latex particles [17]. If anti-*Toxoplasma* antibodies (IgG or IgM or both) are present in the sample, a complex is formed between the T latex and the patient’s antibodies, which is then captured by the T band, and it results in the appearance of a red line (positive test) [17]. The direct capture of the C latex by the C band results in the appearance of a control blue line which indicates that the chromatography performed well [17]. The results are read 20–30 minutes after the eluent solution has been dispensed into the well [17]. Representative example of U.S. sera, negative (left) and positive (right) results. This is the new simple POC test, based on lateral-flow-chromatographic-immunoassay method, already commercially available in France, that detects simultaneously both *Toxoplasma* IgG and IgM antibodies and costs only $4 (the cost we were charged) per test, as opposed to a $650 cost for testing at a commercial laboratory in the U.S.

Despite these present limitations, this test has the potential for true clinical utility in identifying those who need further serologic testing and separating them from those for whom no further screening during pregnancy would be required. In a hypothetical clinical scenario, all pregnant women could be screened at the outset of their visits with an obstetrician or midwife, perhaps even using a multiplexed test for multiple congenital pathogens and *T*. *gondii* IgG and IgM separately [24]. Should the patient’s serum screen positive for *Toxoplasma* IgG/IgM, she could subsequently have confirmatory serologic testing at a reference laboratory for *Toxoplasma* infection performed. Ideally, this would begin at 11 weeks gestation or earlier. If the patient were truly acutely infected in this gestation, based on the results of confirmatory testing at a reference laboratory, she could receive appropriate therapy to reduce the risk of transmission to her fetus as well as the severity of potential clinical disease in the fetus [12, 13]. Should she be identified as chronically infected, she would no longer need further screening for the rest of her gestation. On the other hand, should a patient be negative for both IgG and IgM with the POC test, she could undergo testing on an ongoing, monthly basis during her visits with her obstetric care provider and one month post-partum (to allow also for detection of infections acquired very late in gestation, which nevertheless could have clinical implication for the management of the newborn infant). This will allow rapid detection of seroconversion, as shown with samples obtained in France during seroconversion [17]. Results of the *Toxoplasma* ICT IgG-IgM POC test will progress from negative (Fig 3, left) to positive (Fig 3, right).

This test system will facilitate early identification, diagnosis, and treatment of congenital toxoplasmosis in a cost-saving manner. This approach has been demonstrated to reduce the risk of vertical transmission, as well as the severity of clinical disease in the fetus, and has been demonstrated to be robustly cost-saving in Austria [3, 11–13, 16]. This testing has the capacity to reduce human suffering, be cost-saving for patients and health care systems, and thereby may overcome previous objections to the implementation of screening for congenital toxoplasmosis during pregnancy. It is critical to test such point-of-care tests carefully, as we (FP) also tested another licensed test system, which did not function as well. All rules and regulations pertaining to POC testing will have to be complied with. There is an extremely promising new technique utilizing printed strips for multiplexed tests that can cost US$0.01 (http://med.stanford.edu/news/all-news/2017/02/scientists-develop-lab-on-a-chip-that-costs-1-cent-to-make.html).

There are ongoing studies being performed by our research group, comparing the test studied herein with other POC tests. Some of these point-of-care tests provide separate IgG and IgM results. If some or all of these tests work as well as the *Toxoplasma* ICT IgG-IgM, any, or all, could progress to FDA or other country’s formal approvals and CLIA, or equivalent, certification. Where serologic screening is standard, as it is now in France and Austria, there are economies of scale and possibly other models for testing sites that lower costs. Further, with competing tests, market forces or regulation reduce costs. One cost- benefit analyses provides broadly applicable equations with sensitivity analyses based on outcomes and cost data in the U.S. [15]. Another cost-benefit analysis provides precise, detailed, patient specific, currently accurate data in the very recent analyses from Austria [12]. These types of analyses are complex and beyond the scope of this present work which evaluates the performance of the *Toxoplasma* ICT IgG-IgM with sera from the U.S. and considers the cost and feasibility of implementing such testing.

In countries where obstetrical visits are less frequent than once monthly, monthly gestational testing could incentivize marked improvements in maternal gestational health care and, when multiplexed [24], improve diagnosis of other congenital infections. The information in Fig 2 presents an analysis of possible life and cost savings in a developing country where such screening is implemented monthly during gestation and where urine glucose and blood pressure are also tested during those visits.

## Supporting information

S1 Fig Example of testing with *Toxoplasma* ICT IgG-IgM test and a person whose diagnosis was made long after acquisition during gestation. (TIFF)

S1 Table Infected patient primary data. These samples were chosen to reflect measurement for patients in real time. The samples stored present a unique opportunity to know the precise time from seroconversion (birth of infected infant) to the time the serum sample was obtained. The goal was to determine whether the *Toxoplasma* ICT IgG-IgM POC test result^a^ could distinguish serum from patients infected with parasites of differing serotypes, present in the U.S.^b^, from the sera of uninfected persons. We selected these serum samples at later times after primary infection during gestation that resulted in the birth of an infected infant^c^. The year reflects the time the stored sample was obtained from 1991-2016^d^. The serologic tests in this table were the mother’s serologic tests at the time of the birth of an infected infant^e^. The time after birth the serum was obtained demonstrates that these samples were remote from the primary infection, which is what we were trying to detect. Thirteen samples closer to the time of primary infection were also tested (labeled ^A^). Not all samples from acutely infected persons had serotype data available, reflected by N/A. These data are also displayed in Fig 1. An IgG dye test is considered negative for values <1:16 and positive for values ≥1:16 [26]. An IgM ELISA performed with serum is considered negative for values 0.0–1.6, equivocal for values 1.7–1.9, and positive for values ≥2.0 in serum [27]. An IgM ISAGA is positive for values ≥3 [28]. An IgA ELISA for patients >6 months of age is considered negative for values 0.0–1.4, equivocal for values 1.5–2.0, and positive for values ≥2.1, and an IgA ELISA for patients <6 months of age is considered negative for values 0.0–0.9 and positive for values ≥1.0 [29]. High avidity signifies that infection occurred more than 4 months ago [30]. ^f^ Serology not performed at Palo Alto Medical Foundation *Toxoplasma* Serology Laboratory. ^g^ Chronic seropositive patient. ^h^ Serology values for child are listed. ^i^ Chronic seropositive father; serology values for child are listed. ^H^ Historical control.
(DOCX)

## References

[1] Lykins J, Wang K, Wheeler K, Clouser F, Dixon A, El Bissati K, et al Understanding Toxoplasmosis in the United States Through "Large Data" Analyses. Clin Infect Dis. 2016;63(4):468–75. doi: 10.1093/cid/ciw356 27353665 10.1093/cid/ciw356 PMC4967610

[2] McLeod R, Lee D, Clouser F, Boyer K. Toxoplasmosis in the Fetus and Newborn Infant In: Stevenson DK, Cohen RS, Sunshine P, editors. Neonatology: Clinical Practice and Procedures. 1st ed New York: McGraw Hill; 2015 p. 821–76.

[3] McLeod R, Kieffer F, Sautter M, Hosten T, Pelloux H. Why prevent, diagnose and treat congenital toxoplasmosis? Mem Inst Oswaldo Cruz. 2009;104(2):320–44. 19430661 10.1590/s0074-02762009000200029 PMC2735102

[4] Montoya JG, Remington JS. Management of Toxoplasma gondii infection during pregnancy. Clin Infect Dis. 2008;47(4):554–66. doi: 10.1086/590149 18624630 10.1086/590149

[5] Torgerson PR, Mastroiacovo P. The global burden of congenital toxoplasmosis: a systematic review. Bull World Health Organ. 2013;91(7):501–8. doi: 10.2471/BLT.12.111732 23825877 10.2471/BLT.12.111732 PMC3699792

[6] McPhillie M, Zhou Y, El Bissati K, Dubey J, Lorenzi H, Capper M, et al New paradigms for understanding and step changes in treating active and chronic, persistent apicomplexan infections. Sci Rep. 2016;6:29179 doi: 10.1038/srep29179 27412848 10.1038/srep29179 PMC4944145

[7] El Bissati K, Chentoufi AA, Krishack PA, Zhou Y, Woods S, Dubey JP, et al Adjuvanted multi-epitope vaccines protect HLA-A*11:01 transgenic mice against Toxoplasma gondii. JCI Insight. 2016;1(15):e85955 doi: 10.1172/jci.insight.85955 27699241 10.1172/jci.insight.85955 PMC5033759

[8] Mohle L, Israel N, Paarmann K, Krohn M, Pietkiewicz S, Muller A, et al Chronic Toxoplasma gondii infection enhances beta-amyloid phagocytosis and clearance by recruited monocytes. Acta Neuropathol Commun. 2016;4:25 doi: 10.1186/s40478-016-0293-8 26984535 10.1186/s40478-016-0293-8 PMC4793516

[9] Perry CE, Gale SD, Erickson L, Wilson E, Nielsen B, Kauwe J, et al Seroprevalence and Serointensity of Latent Toxoplasma gondii in a Sample of Elderly Adults With and Without Alzheimer Disease. Alzheimer Dis Assoc Disord. 2016;30(2):123–6. doi: 10.1097/WAD.0000000000000108 26421353 10.1097/WAD.0000000000000108

[10] Ngoungou EB, Bhalla D, Nzoghe A, Darde ML, Preux PM. Toxoplasmosis and epilepsy—systematic review and meta analysis. PLoS Negl Trop Dis. 2015;9(2):e0003525 doi: 10.1371/journal.pntd.0003525 25695802 10.1371/journal.pntd.0003525 PMC4335039

[11] Wallon M, Peyron F, Cornu C, Vinault S, Abrahamowicz M, Kopp CB, et al Congenital toxoplasma infection: monthly prenatal screening decreases transmission rate and improves clinical outcome at age 3 years. Clin Infect Dis. 2013;56(9):1223–31. doi: 10.1093/cid/cit032 23362291 10.1093/cid/cit032

[12] Prusa AR, Kasper DC, Sawers L, Walter E, Hayde M, Stillwaggon E. Congenital toxoplasmosis in Austria: prenatal screening for prevention is cost-saving. PLoS Negl Trop Dis. In press, 2017. 10.1371/journal.pntd.0005648 PMC5503164 28692640

[13] Hotop A, Hlobil H, Gross U. Efficacy of rapid treatment initiation following primary Toxoplasma gondii infection during pregnancy. Clin Infect Dis. 2012;54(11):1545–52. doi: 10.1093/cid/cis234 22460980 10.1093/cid/cis234

[14] Kieffer F, Wallon M, Garcia P, Thulliez P, Peyron F, Franck J. Risk factors for retinochoroiditis during the first 2 years of life in infants with treated congenital toxoplasmosis. Pediatr Infect Dis J. 2008;27(1):27–32. doi: 10.1097/INF.0b013e318134286d 18162934 10.1097/INF.0b013e318134286d

[15] Stillwaggon E, Carrier CS, Sautter M, McLeod R. Maternal serologic screening to prevent congenital toxoplasmosis: a decision-analytic economic model. PLoS Negl Trop Dis. 2011;5(9):e1333 doi: 10.1371/journal.pntd.0001333 21980546 10.1371/journal.pntd.0001333 PMC3181241

[16] Peyron F, Mc Leod R, Ajzenberg D, Contopoulos-Ioannidis D, Kieffer F, Mandelbrot L, et al Congenital Toxoplasmosis in France and the United States: One Parasite, Two Diverging Approaches. PLoS Negl Trop Dis. 2017;11(2):e0005222 doi: 10.1371/journal.pntd.0005222 28207736 10.1371/journal.pntd.0005222 PMC5312802

[17] Chapey E, Wallon M, Peyron F. Evaluation of the LDBIO point of care test for the combined detection of toxoplasmic IgG and IgM. Clin Chim Acta. 2017;464:200–1. doi: 10.1016/j.cca.2016.10.023 27765564 10.1016/j.cca.2016.10.023

[18] McLeod R, Boyer KM, Lee D, Mui E, Wroblewski K, Karrison T, et al Prematurity and severity are associated with Toxoplasma gondii alleles (NCCCTS, 1981–2009). Clin Infect Dis. 2012;54(11):1595–605. doi: 10.1093/cid/cis258 22499837 10.1093/cid/cis258 PMC3348955

[19] Contopoulos-Ioannidis D, Wheeler KM, Ramirez R, Press C, Mui E, Zhou Y, et al Clustering of Toxoplasma gondii Infections Within Families of Congenitally Infected Infants. Clin Infect Dis. 2015;61(12):1815–24. doi: 10.1093/cid/civ721 26405150 10.1093/cid/civ721 PMC4657536

[20] Pomares C, Montoya JG. Laboratory Diagnosis of Congenital Toxoplasmosis. J Clin Microbiol. 2016;54(10):2448–54. doi: 10.1128/JCM.00487-16 27147724 10.1128/JCM.00487-16 PMC5035424

[21] Altman DG, Bland JM. Diagnostic tests. 1: Sensitivity and specificity. BMJ. 1994;308(6943):1552 8019315 10.1136/bmj.308.6943.1552 PMC2540489

[22] Altman DG, Bland JM. Diagnostic tests 2: Predictive values. BMJ. 1994;309(6947):102 8038641 10.1136/bmj.309.6947.102 PMC2540558

[23] Dhakal R, Gajurel K, Pomares C, Talucod J, Press CJ, Montoya JG. Significance of a Positive Toxoplasma Immunoglobulin M Test Result in the United States. J Clin Microbiol. 2015;53(11):3601–5. doi: 10.1128/JCM.01663-15 26354818 10.1128/JCM.01663-15 PMC4609698

[24] Li X, Pomares C, Gonfrier G, Koh B, Zhu S, Gong M, et al Multiplexed Anti-Toxoplasma IgG, IgM, and IgA Assay on Plasmonic Gold Chips: towards Making Mass Screening Possible with Dye Test Precision. J Clin Microbiol. 2016;54(7):1726–33. doi: 10.1128/JCM.03371-15 27008879 10.1128/JCM.03371-15 PMC4922109

[25] Pomares C, Zhang B, Arulkumar S, Gonfrier G, Marty P, Zhao S, et al Validation of IgG, IgM multiplex plasmonic gold platform in French clinical cohorts for the serodiagnosis and follow-up of Toxoplasma gondii infection. Diagn Microbiol Infect Dis. 2017;87(3):213–8. doi: 10.1016/j.diagmicrobio.2016.09.001 28040304 10.1016/j.diagmicrobio.2016.09.001

[26] Sabin AB, Feldman HA. Dyes as Microchemical Indicators of a New Immunity Phenomenon Affecting a Protozoon Parasite (Toxoplasma). Science. 1948;108(2815):660–3. doi: 10.1126/science.108.2815.660 17744024 10.1126/science.108.2815.660

[27] Naot Y, Remington JS. An enzyme-linked immunosorbent assay for detection of IgM antibodies to Toxoplasma gondii: use for diagnosis of acute acquired toxoplasmosis. J Infect Dis. 1980;142(5):757–66. 7007520 10.1093/infdis/142.5.757

[28] Desmonts G, Naot Y, Remington JS. Immunoglobulin M-immunosorbent agglutination assay for diagnosis of infectious diseases: diagnosis of acute congenital and acquired Toxoplasma infections. J Clin Microbiol. 1981;14(5):486–91. 7031082 10.1128/jcm.14.5.486-491.1981 PMC273974

[29] Stepick-Biek P, Thulliez P, Araujo FG, Remington JS. IgA antibodies for diagnosis of acute congenital and acquired toxoplasmosis. J Infect Dis. 1990;162(1):270–3. 2192008 10.1093/infdis/162.1.270

[30] Pelloux H, Brun E, Vernet G, Marcillat S, Jolivet M, Guergour D, et al Determination of anti-Toxoplasma gondii immunoglobulin G avidity: adaptation to the Vidas system (bioMerieux). Diagn Microbiol Infect Dis. 1998;32(2):69–73. 9823527 10.1016/s0732-8893(98)00077-7

