# Supplementary figures and images for: Point-of-care testing for Toxoplasma gondii IgG/IgM using Toxoplasma ICT IgG-IgM test with sera from the United States and implications for developing countries
Source: PLoS Negl Trop Dis. 2017 Jun 26;11(6):e0005670. doi: 10.1371/journal.pntd.0005670 (PMC5501679; doi:10.1371/journal.pntd.0005670)

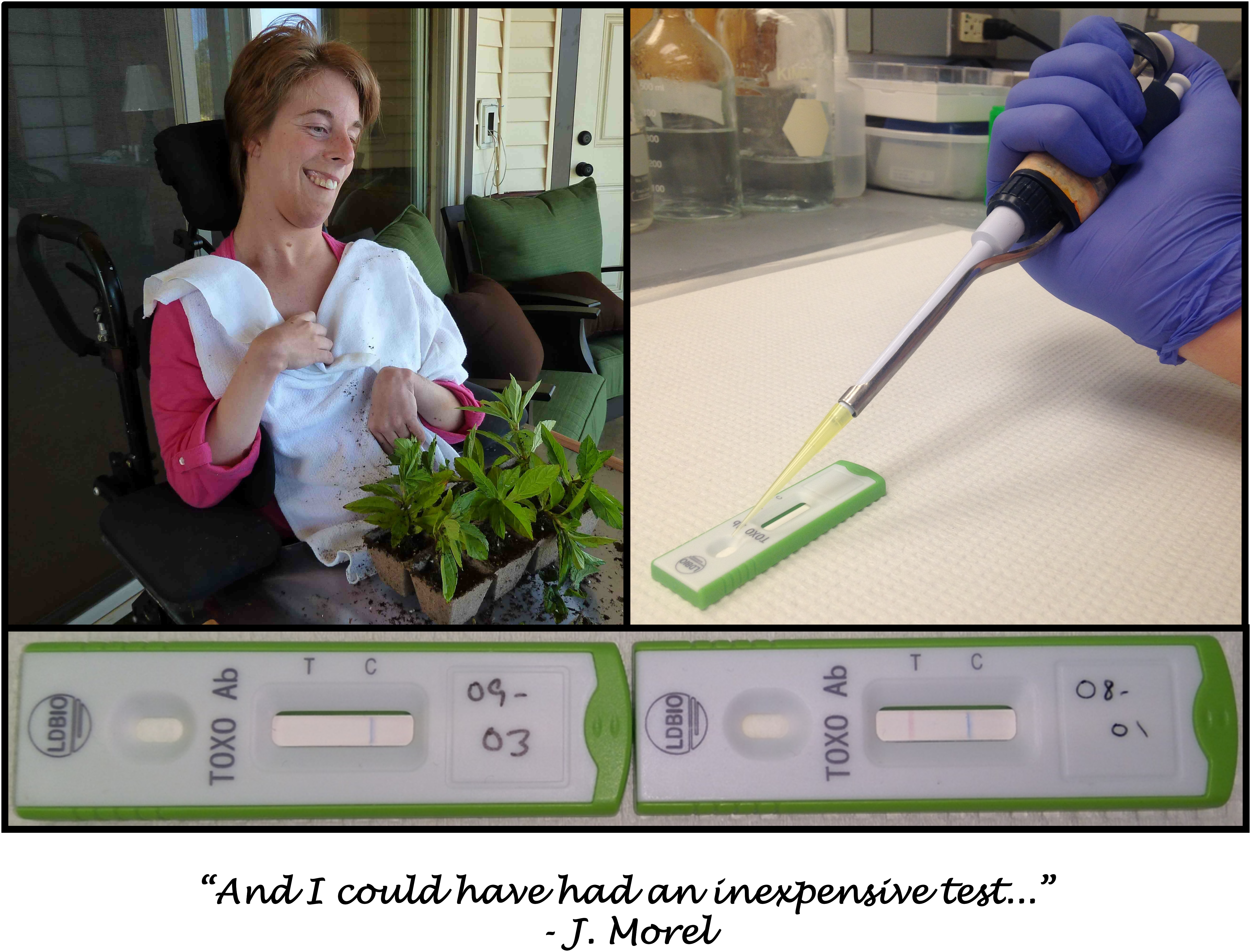

Supplement: S1 Fig — (TIFF) [file pntd.0005670.s001.tiff]
