# Supplementary material for: Point-of-care testing for Toxoplasma gondii IgG/IgM using Toxoplasma ICT IgG-IgM test with sera from the United States and implications for developing countries
Source: PLoS Negl Trop Dis. 2017 Jun 26;11(6):e0005670. doi: 10.1371/journal.pntd.0005670 (PMC5501679; doi:10.1371/journal.pntd.0005670)
Supplement: S1 Table — These samples were chosen to reflect measurement for patients in real time. The samples stored present a unique opportunity to know the precise time from seroconversion (birth of infected infant) to the time the serum sample was obtained. The goal was to determine whether the Toxoplasma ICT IgG-IgM POC test resulta could distinguish serum from patients infected with parasites of differing serotypes, present in the U.S.b, from the sera of uninfected persons. We selected these serum samples at later times after primary infection during gestation that resulted in the birth of an infected infantc. The year reflects the time the stored sample was obtained from 1991-2016d. The serologic tests in this table were the mother’s serologic tests at the time of the birth of an infected infante. The time after birth the serum was obtained demonstrates that these samples were remote from the primary infection, which is what we were trying to detect. Thirteen samples closer to the time of primary infection were also tested (labeled A). Not all samples from acutely infected persons had serotype data available, reflected by N/A. These data are also displayed in Fig 1. An IgG dye test is considered negative for values <1:16 and positive for values ≥1:16 [26]. An IgM ELISA performed with serum is considered negative for values 0.0–1.6, equivocal for values 1.7–1.9, and positive for values ≥2.0 in serum [27]. An IgM ISAGA is positive for values ≥3 [28]. An IgA ELISA for patients >6 months of age is considered negative for values 0.0–1.4, equivocal for values 1.5–2.0, and positive for values ≥2.1, and an IgA ELISA for patients <6 months of age is considered negative for values 0.0–0.9 and positive for values ≥1.0 [29]. High avidity signifies that infection occurred more than 4 months ago [30]. f Serology not performed at Palo Alto Medical Foundation Toxoplasma Serology Laboratory. g Chronic seropositive patient. h Serology values for child are listed. i Chronic seropositive father; serol [file pntd.0005670.s002.docx]

**Supporting Information**

**Point-of-care testing for *Toxoplasma gondii* IgG/IgM using *Toxoplasma* ICT IgG-IgM test with sera from the United States and implications for developing countries**

Ian J. Begeman^1¶^, Joseph Lykins^2¶^, Ying Zhou^1^, Bo Shiun Lai^1^, Pauline Levigne^3^, Kamal El Bissati^1^, Kenneth Boyer^1,4^, Shawn Withers^1^, Fatima Clouser^1^, A. Gwendolyn Noble^1,5^, Peter Rabiah^1,6^, Charles N. Swisher^1,5^, Peter T. Heydemann^1,4^, Despina G. Contopoulos-Ioannidis^7,8^, Jose G. Montoya^8,9^, Yvonne Maldonado^7^, Raymund Ramirez^8^, Cindy Press^8^, Eileen Stillwaggon^10^, François Peyron^3^, Rima McLeod^1,11*^

^1^ Department of Ophthalmology and Visual Science, The University of Chicago, Chicago, IL, USA

^2^ Pritzker School of Medicine, The University of Chicago, Chicago, IL, USA

^3^ Institut de Parasitologie et de Mycologie Médicale Hôpital de la Croix Rousse, 103 grande rue de la Croix Rousse, 69317, Lyon, France

^4^ Rush University and Medical Center, Chicago, IL, USA

^5^ Lurie Children’s Hospital and Northwestern University, Chicago, IL, USA

^6^ Northshore Hospital, Evanston, IL, USA

^7^ Department of Pediatrics, Division of Infectious Diseases, Stanford University School of Medicine, Stanford, CA, USA

^8^ Palo Alto Medical Foundation *Toxoplasma* Serology Laboratory, Palo Alto, CA, USA

^9^ Department of Medicine, Division of Infectious Diseases and Geographic Medicine, Stanford University School of Medicine, Stanford, CA, USA

^10^ Gettysburg College, Gettysburg, PA, USA

^11^ Department of Pediatrics (Infectious Diseases), Institute of Genomics, Genetics, and Systems Biology, Global Health Center, Toxoplasmosis Center, the Center for Health and the Social Sciences, CHeSS, the College, The University of Chicago, Chicago Medicine, Chicago IL, USA

^*^ Corresponding author

E-mail: rmcleod@uchicago.edu

^¶^ These authors contributed equally to this work.

## S1 Table. Infected patient primary data.

| ***Toxoplasma* ICT**  **IgG-IgM Test Result (Test/Control)^a^** | **Parasite Serotype/Cohort^b^** | **Days from Birth to Sample Collection^c^** | **Year Sample Obtained^d^** | **Reciprocal IgG^e^** | **IgM^e^** | **IgA^e^** | **Avidity^e^** | **AC/HS^e^** |
| --- | --- | --- | --- | --- | --- | --- | --- | --- |
| Positive/Positive | Atypical | 2372^H^ | 1991 | 128 | ELISA: 1.1 | 0.6 | ND | 50/800 |
| Positive/Positive | Atypical | 83^A^ | 1993 | 2048 | ELISA: 4 | 11.9 | ND | 400/3200 |
| Positive/Positive | Atypical | 481 | 1993 | 4096 | ELISA: 2.9 | 9.7 | ND | 1600/3200 |
| Positive/Positive | Atypical | 551 | 1994 | 8000 | ELISA: 2.6 | 1.8 | ND | 400/3200 |
| Positive/Positive | Atypical^A^ | -2 | 1995 | 1024 | ELISA: 8.7 | 4.9 | ND | 400/100 |
| Positive/Positive | Atypical | 69^A^ | 1995 | 4096 | ELISA: 4.4 | 9.6 | ND | 1600/3200 |
| Positive/Positive | Atypical | 3874 | 1995 | 16000 | ELISA: 2.8 | ND | ND | ND |
| Positive/Positive | Atypical | 112 | 1995 | 4096 | ELISA: 5.0 | ND | ND | 1600/3200 |
| Positive/Positive | Atypical | 1401 | 1995 | 8000 | ELISA: 5.4 | 22.4 | ND | 1600/3200 |
| Positive/Positive | Atypical | 3720 | 1996 | 8000 | ELISA: 0.7 | ND | ND | ND |
| Positive/Positive | Atypical | 1286^H^ | 1996 | 512 | ELISA: .2 | 0.5 | ND | 50/800 |
| Positive/Positive | Atypical | 0^A^ | 1997 | 8000 | ELISA: 7.4 | 1.3 | ND | 200/3200 |
| Positive/Positive | Atypical | 1308 | 1997 | 4096 | ISAGA: 12 | 10.7 | ND | 1600/3200 |
| Positive/Positive | Atypical | 910 | 1998 | 2048 | 5.7 | 1.2 | ND | 50/1600 |
| Positive/Positive | Atypical | 2150 | 2003 | 8000 | ELISA: 7.4 | 1.3 | ND | 200/3200 |
| Positive/Positive | Atypical | 5293 | 2004 | 4096 | ELISA: 3.8 | 5.3 | ND | 800/3200 |
| Positive/Positive | Atypical | 3828 | 2005 | 16000 | ELISA: 9.4 | 17.6 | ND | 1600/1600 |
| Positive/Positive | Atypical | 1519 | 2007 | 16000 | ND | ND | ND | ND |
| Positive/Positive | Atypical | 5455^H^ | 2008 | 512 | ELISA: 0.2 | 1.0 | ND | ND |
| Positive/Positive | Atypical | 5716 | 2008 | 4096 | ELISA: 3.8 | 11.4 | ND | 1600/3200 |
| Positive/Positive | Atypical | 6280 | 2008 | 1024 | ELISA: 9.3 | 10.8 | ND | 1600/3200 |
| Positive/Positive | Atypical | 9137 | 2008 | 1024 | ELISA: 6.9 | ND | ND | ND |
| Positive/Positive | Atypical | 5466 | 2009 | 8000 | ISAGA: 12 | 18.1 | ND | 1600/3200 |
| Positive/Positive | Atypical | 3524 | 2009 | 8000 | ELISA: 2.3 | 9.1 | ND | 1600/3200 |
| Positive/Positive | Atypical | 7683 | 2009 | 2048 | ELISA: 3.1 | ND | ND | 1600/3200 |
| Positive/Positive | Atypical | 3605 | 2010 | 16000 | ELISA: 9.1 | 9.5 | ND | 1600/3200 |
| Positive/Positive | Atypical | 6976 | 2010 | 8000 | ELISA: 10.8 | 2.4 | ND | 1600/3200 |
| Positive/Positive | Atypical | 1955 | 2010 | 16000 | ELISA: 10 | ND | Low | 1600/3200 |
| Positive/Positive | Atypical | 5698 | 2011 | 4096 | ELISA: 5.6 | 22.4 | ND | ND |
| Positive/Positive | Atypical | 2666 | 2011 | 2048 | Negative | Negative | ND | 200/1600 |
| Positive/Positive | Atypical | 5397 | 2012 | 32000 | ELISA: 9.1 | 1.5 | ND | 1600/3200 |
| Positive/Positive | Atypical | 9170^H^ | 2016 | 2048 | ELISA: 0 | 0.7 | ND | 50/3200 |
| Positive/Positive | I/III | 1585 | 1992 | 4096 | ELISA: 3.4 | ND | ND | ND |
| Positive/Positive | I/III | 145 | 1993 | 2048 | ELISA: 5.6 | 12.4 | ND | 1600/3200 |
| Positive/Positive | I/III | 1880^H^ | 1997 | 2048 | ELISA: 2.6 | 2.5 | ND | 200:3200 |
| Positive/Positive | I/III | 5362^H^ | 1997 | 2048 | ELISA: 0.6 | 0.7 | ND | 50/200 |
| Positive/Positive | I/III | 59^A^ | 2001 | 16000 | ELISA: 7.8 | 14.4 | ND | 1600/800 |
| Positive/Positive | I/III | 3453 | 2004 | 4096 | ELISA: 2.6 | ND | ND | 1600/3200 |
| Positive/Positive | I/III | 1246 | 2004 | 16000 | ELISA: 7.8 | 14.4 | ND | 1600/800 |
| Positive/Positive | I/III | 2800 | 2005 | 3000^f^ | ISAGA: 12^f^ | ND | ND | ND |
| Positive/Positive^g^ | I/III^g^ | 22519^g,H^ | 2008^g^ | 16^g^ | ELISA: 0^g^ | 0^g^ | ND^g^ | 50/200^g^ |
| Positive/Positive | I/III | 636 | 2009 | 128 | ELISA:2.1 | ND | Low | <50/<100 |
| Positive/Positive | I/III | 7961 | 2009 | 8000 | ELISA: 10.9 | ND | ND | 400/3200 |
| Positive/Positive | I/III | 6494 | 2011 | 16000 | ELISA: 10.2 | 10.9 | ND | 1600/3200 |
| Positive/Positive | I/III | 1858 | 2012 | 4096 | ELISA: 5.4 | ND | ND | ND |
| Positive/Positive | I/III | 1949 | 2012 | 32000 | ELISA: 6.1 | 11.2 | ND | 1600/3200 |
| Positive/Positive | I/III | 7482^H^ | 2013 | 512 | ELISA: 0.0 | 0.4 | ND | 200/800 |
| Positive/Positive | I/IIIa | 558 | 1990 | 2048 | ELISA: 8.4 | ND | ND | 1600/3200 |
| Positive/Positive | I/IIIa | 856 | 1992 | 8000 | ELISA: 5.5 | 8.5 | ND | 1600/1600 |
| Positive/Positive | I/IIIa | 3798 | 2004 | 4096 | ISAGA: 12 | 8.2 | ND | 1600/3200 |
| Positive/Positive | I/IIIa | 4560^H^ | 2005 | 1024 | ELISA: 0.1 | 0.1 | ND | 50/3200 |
| Positive/Positive | I/IIIa | 1721 | 2005 | 8000 | ELISA: 8.1 | 6.2 | ND | 1600/1600 |
| Positive/Positive | I/IIIa | 7331^H^ | 2006 | 8000 | ELISA: 1.2 | ND | ND | ND |
| Positive/Positive | I/IIIa | 5210^H^ | 2007 | 1024 | ELISA: 3.3 | 1.6 | ND | 100/800 |
| Positive/Positive | I/IIIa | 3657 | 2007 | 2048 | ELISA: 1.6 | 0.2 | ND | 50/3200 |
| Positive/Positive | I/IIIa | 5435 | 2008 | 128^h^ | ISAGA: 11^h^ | 3^h^ | ND^h^ | 1600/3200^h^ |
| Positive/Positive | I/IIIa | 5350 | 2009 | 8000 | ELISA: 2.8 | 2.6 | ND | 1600/3200 |
| Positive/Positive | I/IIIa | 1662 | 2009 | 2048 | ND | ND | Low | 400/800 |
| Positive/Positive | I/IIIa | 7197 | 2009 | 8000 | ELISA: 0.2 | 0.2 | ND | 400/3200 |
| Positive/Positive | I/IIIa | 7250 | 2010 | 4096 | ELISA: 10.9 | 7.6 | ND | 200/800 |
| Positive/Positive | I/IIIa | 8563 | 2010 | 4096 | ELISA: 4.6 | ND | ND | ND |
| Positive/Positive | I/IIIa | 7265 | 2010 | 8000 | ELISA: 8.3 | 8.1 | ND | 1600/3200 |
| Positive/Positive | I/IIIa | 3447 | 2011 | 16000 | ELISA: 2.6 | 20.1 | ND | 800/3200 |
| Positive/Positive | I/IIIa | 3689 | 2011 | 8000 | ELISA: 3.4 | 22.2 | ND | 1600/3200 |
| Positive/Positive | I/IIIa | 5334 | 2011 | 32000 | ELISA: 10.5 | 24 | ND | 1600/3200 |
| Positive/Positive | I/IIIa | 4506^H^ | 2011 | 512 | ELISA: 0.2 | 1.6 | ND | 50/800 |
| Positive/Positive | I/IIIa | 5497 | 2011 | 32000 | ELISA: 9.2 | 35 | ND | 1600/3200 |
| Positive/Positive | I/IIIa | 6701 | 2012 | 4096 | ISAGA: 12 | 10.8 | ND | 1600/3200 |
| Positive/Positive | I/IIIa | 7296 | 2012 | 4096 | ELISA: 1.8 | 14.7 | ND | 1600/3200 |
| Positive/Positive | I/IIIa | 3934 | 2013 | 8000 | ELISA: 10 | ND | ND | ND |
| Positive/Positive | I/IIIa | 2740 | 2014 | 4096 | ELISA: 5.4 | ND | ND | ND |
| Positive/Positive | I/IIIa | 3024 | 2015 | 4096 | ELISA: 9.6 | ND | ND | ND |
| Positive/Positive | II | 1097 | 1987 | 2048 | ELISA: 6.3 | ND | ND | ND |
| Positive/Positive | II | 53^A^ | 1990 | 256 | ELISA: 3.9 | 2.8 | ND | 200/100 |
| Positive/Positive | II | 4382^H^ | 1994 | 512 | ELISA: 1.3 | ND | ND | ND |
| Positive/Positive | II | 2819 | 1994 | 2048 | ELISA: 1.1 | ND | ND | ND |
| Positive/Positive | II | 64^A^ | 1994 | 16000 | ELISA: 3.3 | 22.2 | ND | 1600/3200 |
| Positive/Positive | II | 366 | 1994 | 1024 | ELISA: 6.7 | 5.8 | ND | 1600/3200 |
| Positive/Positive | II | 2762^H^ | 1995 | 256 | ELISA: 3.8 | ND | ND | 50/800 |
| Positive/Positive | II | 3667 | 1995 | 4096 | ELISA: 1.5 | 2.3 | ND | ND |
| Positive/Positive | II | 382 | 1995 | 512 | ELISA: 0 | 1.1 | ND | 800/1600 |
| Positive/Positive | II | 8696^H^ | 1998 | 4 | ND | ND | ND | 50/200 |
| Positive/Positive | II | 4558^H^ | 1998 | 512 | ELISA: 0.4 | 0.4 | ND | 50/200 |
| Positive/Positive | II | 247 | 2000 | 16000 | ELISA: 1.6 | ND | ND | 1600/3200 |
| Positive/Positive | II | 3702 | 2002 | 2048 | ELISA: 3 | 2.3 | ND | 400/3200 |
| Positive/Positive | II | 6196 | 2003 | 8000 | ELiSA: 4.5 | ND | ND | ND |
| Positive/Positive | II | 3825 | 2003 | 2048 | ELISA: 2.1 | 0.8 | ND | 800/3200 |
| Positive/Positive | II | 4110 | 2003 | 4096 | ELISA: 6.7 | 5.6 | ND | 1600/3200 |
| Positive/Positive | II | 3598 | 2003 | 2048 | ELISA: 5.4 | 7.9 | ND | 1600/3200 |
| Positive/Positive | II | 45^A^ | 2004 | 2048 | ELISA: 2.5 | 2.3 | ND | ND |
| Positive/Positive | II | 17^A^ | 2005 | 8000 | ELISA: 1.5 | Negative | 2.1 low | 400/800 |
| Positive/Positive | II | 51^A^ | 2005 | 2048 | ELISA: 5.2 | 0.8 | Low | 400/400 |
| Positive/Positive | II | 5420^H^ | 2006 | 2048 | 1.5 | ND | ND | 400/3200 |
| Positive/Positive | II | 376 | 2006 | 8000^h^ | ISAGA: Negative^h^ | 0.7^h^ | ND^h^ | ND^h^ |
| Positive/Positive | II | 388 | 2006 | 8000 | ELISA: 1.5 | Negative | 2.1 low | 400/800 |
| Positive/Positive | II | 6585 | 2006 | 128 | ELISA: 7.2 | ND | ND | 50/400 |
| Positive/Positive | II | 5380 | 2007 | 4096 | ELISA: 5.2 | 6.9 | ND | 1600/3200 |
| Positive/Positive | II | 7293 | 2007 | 8000 | ELISA: 2 | ND | ND | ND |
| Positive/Positive | II | 375 | 2007 | 512 | ELISA: 8.7 | 3.8 | ND | 50/100 |
| Positive/Positive | II | 7754^H^ | 2008 | 256 | ELISA: 0 | 1 | ND | 50/400 |
| Positive/Positive | II | 7595^H^ | 2008 | 1024 | ELISA: 1.6 | 3.2 | ND | 400/3200 |
| Positive/Positive | II | 7098^H^ | 2009 | 128 | ELISA: 0.5 | ND | ND | ND |
| Positive/Positive | II | 7206^H^ | 2009 | 256 | ELISA: 2.8 | ND | ND | ND |
| Positive/Positive | II | 3667 | 2009 | 2048 | ELISA: 5.0 | 0.8 | ND | ND |
| Positive/Positive | II | 3568 | 2009 | 32000 | ELISA: 7.1 | 28 | ND | 1600/1600 |
| Positive/Positive | II | 5559 | 2010 | 8000 | ISAGA: 7 | 3.5 | ND | 400/3200 |
| Positive/Positive | II | 7367^H^ | 2010 | 128 | ELISA: 0.8 | ND | ND | ND |
| Positive/Positive | II | 8504 | 2010 | 16000 | ELISA: 1.6 | ND | ND | ND |
| Positive/Positive | II | 2242 | 2011 | 2048 | ELISA: 5.2 | 0.8 | Low | 400/400 |
| Positive/Positive | II | 4697 | 2011 | 512 | ELISA: 4.1 | 3.7 | ND | 200/800 |
| Positive/Positive | II | 3750 | 2011 | 16000 | ELISA: 2.8 | 1.2 | ND | 1600/3200 |
| Positive/Positive | II | 2204 | 2011 | 2048 | ELISA: 3.1 | 0.7 | ND | 800/800 |
| Positive/Positive | II | 7192 | 2011 | 2048 | ELISA: 3.7 | 2.1 | ND | 1600/3200 |
| Positive/Positive | II | 3548 | 2012 | 8000 | ELISA: 2.9 | 1.8 | High | 1600/800 |
| Positive/Positive | II | 3013 | 2012 | 4096 | ELISA: 8.5 | 1 | Low | 1600/800 |
| Positive/Positive | II | 6113 | 2012 | 2048 | ELISA: 1.4 | 0.6 | ND | 400/800 |
| Positive/Positive | II | 9515 | 2013 | 2048 | ELISA: 3.5 | ND | ND | ND |
| Positive/Positive | II | 7480^H^ | 2013 | 1024 | ND | ND | ND | ND |
| Positive/Positive | II | 8203 | 2013 | 1024 | ELISA: 3.3 | 5.3 | ND | 1600/800 |
| Positive/Positive | II | 1863 | 2013 | 8000^h^ | ISAGA: Positive^h^ | 11.1^h^ | ND^h^ | ND^h^ |
| Positive/Positive | II | 9718 | 2013 | 4096 | ELISA: 2.8 | ND | ND | ND |
| Positive/Positive | II | 3715 | 2013 | 16000 | ELISA: 3.2 | 10.5 | Low | 800/1600 |
| Positive/Positive | II | 7654 | 2014 | 16000 | ELISA: 8 | 4.3 | ND | 1600/3200 |
| Positive/Positive | II | 3088 | 2014 | 8000 | ELISA: 1.7 | 1.5 | ND | 1600/3200 |
| Positive/Positive | II | 4281 | 2016 | 2048 | ELISA: 2.5 | 2.3 | ND | ND |
| Positive/Positive | Type not known | 8^A^ | 2006 | 2048 | ELISA: 3.5 | ND | ND | 800/800 |
| Positive/Positive | Type not known | 50^A^ | 2009 | 8000 | ELISA: 3 | 3.5 | Equivocal | 1600/3200 |
| Positive/Positive^i^ | Type not known^i^ | 445^i^ | 2010^i^ | 16000^i^ | ISAGA: Positive^i^ | 8.5^i^ | ND^i^ | ND^i^ |
| Positive/Positive | Type not known | 57^A^ | 2011 | 32000 | ELISA: 0.1 | Negative | Equivocal | 800/3200 |

These samples were chosen to reflect measurement for patients in real time. The samples stored present a unique opportunity to know the precise time from seroconversion (birth of infected infant) to the time the serum sample was obtained. The goal was to determine whether the *Toxoplasma* ICT IgG-IgM POC test result^a^ could distinguish serum from patients infected with parasites of differing serotypes, present in the U.S.^b^, from the sera of uninfected persons. We selected these serum samples at later times after primary infection during gestation that resulted in the birth of an infected infant^c^. The year reflects the time the stored sample was obtained from 1991-2016^d^. The serologic tests in this table were the mother’s serologic tests at the time of the birth of an infected infant^e^. The time after birth the serum was obtained demonstrates that these samples were remote from the primary infection, which is what we were trying to detect. Thirteen samples closer to the time of primary infection were also tested (labeled ^A^). Not all samples from acutely infected persons had serotype data available, reflected by N/A. These data are also displayed in Fig 1. An IgG dye test is considered negative for values <1:16 and positive for values ≥1:16 [27]. An IgM ELISA performed with serum is considered negative for values 0.0-1.6, equivocal for values 1.7-1.9, and positive for values ≥2.0 in serum [28]. An IgM ISAGA is positive for values ≥3 [29]. An IgA ELISA for patients >6 months of age is considered negative for values 0.0-1.4, equivocal for values 1.5-2.0, and positive for values ≥2.1, and an IgA ELISA for patients <6 months of age is considered negative for values 0.0-0.9 and positive for values ≥1.0 [30]. High avidity signifies that infection occurred more than 4 months ago [31]. ^f^ Serology not performed at Palo Alto Medical Foundation *Toxoplasma* Serology Laboratory. ^g^ Chronic seropositive patient. ^h^ Serology values for child are listed. ^i^ Chronic seropositive father; serology values for child are listed. ^H^ Historical control.
